# Involvement of neurokinin-1 receptors in the autonomic nervous system in colorectal distension-induced cardiovascular suppression in rats

**DOI:** 10.3389/fphar.2022.1020685

**Published:** 2022-10-19

**Authors:** Kentaro Kurogochi, Masami Uechi, Kensuke Orito

**Affiliations:** ^1^ Laboratory of Physiology II Department of Veterinary Medicine, School of Veterinary Medicine, Azabu University, Sagamihara, Kanagawa, Japan; ^2^ JASMINE Veterinary Cardiovascular Medical Center, Yokohama, Kanagawa, Japan

**Keywords:** c-Fos, fosaprepitant, tachykinin neurokinin-1 receptor, vagal reflex, colorectal distention, autonomic nervous system

## Abstract

Situational syncope, which includes rectally mediated reflexes, is defined as syncope induced by a specific situation. Its pathogenesis generally involves disorders of the autonomic nervous system. However, the mechanisms and preventive strategies are not yet well understood. Therefore, we hypothesized that a tachykinin neurokinin-1 receptor might be involved in the autonomic nervous system, and that a neurokinin-1 receptor antagonist could mitigate reflex syncope. This study used a rat model in which the reflex was induced by afferent vagal stimulation with colorectal distension (CRD). In the study, the rats were divided into three groups: non-CRD, CRD, and CRD with a neurokinin-1 receptor antagonist. First, we examined the effect of fosaprepitant, a neurokinin-1 receptor antagonist, on the circulatory response in this model. We then determined the brain regions that showed increased numbers of c-Fos immunoreactive cells in the respective groups. Our results suggest that the colorectal distension procedure reduced blood pressure and that fosaprepitant lowered this response. In addition, the number of c-Fos immunoreactive cells was increased in the caudal ventrolateral medullary region with colorectal distension, and this number was decreased by the administration of fosaprepitant. In conclusion, fosaprepitant might be involved in the vagal reflex pathway and potentially suppress the circulatory response to colorectal distension.

## Introduction

Situational syncope is defined as syncope induced by a specific situation. Its pathogenesis generally involves a disorder of the autonomic nervous system: a rapid increase in vagal activity, decrease in sympathetic activity, and decrease in cardiac preload. Situational syncope includes fainting attacks caused by micturition, swallowing, coughing, breath-holding (Valsalva maneuver), and vomiting, as reported in humans and dogs (Brignole et al., 2018; Fukushima et al., 2018; Santilli et al., 2019).

Reflexes associated with the rectum also cause disorders of the autonomic nervous system, such as vagal hyperactivity and sympathetic inhibition of stimulus receptors in the gastrointestinal tract. Rectally mediated reflexes have been reported to occur during defecation syncope (Bae et al., 2012), transrectal ultrasound-guided prostate biopsy (Kölükçü et al., 2019), computed tomographic colonography (Neri et al., 2007), and colostomy irrigation (Sadahiro et al., 1995). However, the central nervous system pathways and preventive strategies for these problems have not yet been well established.

Although the stimulus mechanoreceptors differ for each type of syncope, the reflex pathways of the autonomic nervous system are similar. In brief, vagal afferents terminate in the nucleus of the solitary tract, which sends primary projections to the caudal ventrolateral medullary region. In addition, the rostral ventrolateral medullary region, which integrates cardiovascular motion, receives inhibitory projections from the caudal ventrolateral medulla (Reis et al., 1984; Holstein et al., 2012). These central pathways are considered to be involved in the development of bradycardia through vagal hyperactivity and hypotension through sympathetic inhibition.

The neurokinin-1 receptor is a member of the seven-transmembrane G protein-coupled receptor family and induces activation of phospholipase C to produce inositol triphosphate (Guard and Watson, 1991) in the central nervous system. It plays a role in a wide range of physiological functions and disease pathophysiology (Maggi, 1995), including pain, neurogenic inflammation, and emotion, and may also be involved in regulating the autonomic nervous system (Mistrova et al., 2016).

As reported previously, neurons with neurokinin-1 receptor immunoreactivity were found ventral to the nucleus ambiguus, the nucleus of the solitary tract, and the ventromedial medulla, and some neurons close to the rostral compact portion of the nucleus ambiguus (Wang et al., 2001). In summary, neurokinin-1 receptor immunoreactivity is present in many types of ventral medullary neurons. We hypothesized, therefore, that neurokinin-1 receptors might be involved in the autonomic nervous system, and that a neurokinin-1 receptor antagonist would have the potential to mitigate reflex syncope.

Colorectal distension induces the vagal reflex by afferent vagal stimulation in a rat model (Li et al., 2006). It also induces c-Fos in the lumbosacral spinal cord as a noxious visceral stimulus (Traub et al., 1992), which could be repressed by anesthesia and analgesia (Ness and Gebhart, 1988; Traub et al., 1995). We used a urethane-anesthetized rat model in which the vagal reflex was induced by a stimulation with colorectal distension and investigated whether this response could be suppressed using a neurokinin-1 receptor antagonist. We believe that this research may elucidate the mechanism of the situational reflexes and the establishment of treatment for them.

## Materials and methods

### Animals

Animal care and handling followed the Azabu University Animal Experiment Guidelines. All experiments were approved by the Committee for Animal Experimentation at Azabu University (approval number: 191205-8). Data from 18 Wistar rats (SLC, Shizuoka, Japan), weighing 250–350 g, were used in this study. Three rats were housed in a cage (225 mm^3^ × 338 mm^3^ × 140 mm^3^) at 23 ± 2°C and 60% ± 10% humidity under a 12-h light-dark schedule (lights on from 7:00 to 19:00) and provided water and regular food pellets (CREA Rodent Diet CE-2^®^; CREA Japan Inc., Tokyo, Japan) *ad libitum*. All animal experiments were initiated after an acclimation period of at least 1 week. Eighteen rats were randomly divided into two treatment groups and a control group. The treatment groups with colorectal distension were administered saline (Group CRD) or a neurokinin-1 receptor antagonist (Group CRD + NK_1_A). A control group without colorectal distension was also prepared for histological evaluation.

### Procedure of colorectal distension

The colorectal distension procedure was performed with modifications to a technique based on previous research (Li et al., 2006). The rats were anesthetized with 1.2 g/kg urethane intraperitoneally. As we had difficulties obtaining hemodynamic stabilization with other anesthetics in a preliminary experiment, we deemed urethane essential in this study to capture subtle changes in circulatory dynamics. Its preparation, use, and storage were strictly controlled according to guidelines (Guidelines for Urethane, UC San Diego, 2016^1^). To prepare for the experiment, catheters were placed in the femoral artery, and mean arterial pressure and heart rate were recorded. The trachea was cannulated, and body temperature was maintained using a thermal mat and blanket. The animals were kept in the supine position during the experiment. After preparation, either 30 mg/kg of fosaprepitant meglumine (PROEMEND^®^, Ono Pharmaceutical Co., LTD., Osaka, Japan), a prodrug of aprepitant, or saline was administered to each group intraperitoneally. The total volume of drugs was adjusted to 10 ml/kg by dilution with saline. The fosaprepitant dose was set based on an earlier study (Prasoon et al., 2016).

The colorectal distension procedure was started 40 min after drug administration. Colorectal distension was induced by inserting a 2.0 cm air-filled balloon made from a latex glove into the colorectum through the anus; the tip of the balloon was placed at a depth of 6.0 cm. The degree of distension was assessed using a syringe filled with room air and a pressure transducer connected to the balloon by a T-shaped cock. Balloon pressures of 40, 60, and 80 mmHg were applied for 60 s, and intervals between distension were approximately 5 min.

Measurements were made using a pressure transducer (DX-300, Nihon Kohden, Tokyo, Japan) and converted into digital signals *via* an analog-to-digital converter for analysis. The maximum changes in mean arterial pressure recorded during colorectal distension stimulation were compared with the pre-stimulus control values.

### Tissue fixation, sectioning, and staining

Rats for the present study were perfused transcardially with 100 ml of (37°C) 10 mM phosphate-buffered saline followed by 500 ml of 4% paraformaldehyde fixative in 0.1 M phosphate-buffered saline (pH 7.4) at room temperature for 90 min after the completion of the colorectal distension. The brains were removed, harvested at 4°C for 2 days, and stored at 4°C in phosphate-buffered saline until histological processing was performed.

Tissue blocks were cut using a vibratome (Microslicer DTK-1000, Dosaka, Kyoto, Japan) into 50 μm thick serial sections that extended through the entire medulla oblongata.

After inactivation of endogenous peroxidase in 10 mM phosphate-buffered saline containing 3% hydrogen peroxide for 20 min and washing in phosphate-buffered saline (3 min × 5 min), the sections were transferred to a blocking solution (phosphate-buffered saline containing 1% normal goat serum) for 1 h.

The avidin-biotinylated peroxidase complex method was performed with modifications based on a previous study (Pete et al., 2002; Kakiuchi et al., 2014). First, the sections were incubated in a blocking solution containing a primary antibody (polyclonal rabbit anti-c-Fos; Santa Cruz Biotechnology, Santa Cruz, CA, United States) at a 1:10,000 dilution at room temperature for 24 h. Next, the primary antibody was washed in phosphate-buffered saline (3 min × 5 min), and the sections were incubated with the secondary antibody (biotinylated IgG; Vector Laboratories, Burlingame, CA, United States) diluted in a blocking solution (1:200) for 90 min. After washing the secondary antibody in phosphate-buffered saline (3 min × 5 min), the sections were subjected to an avidin/biotin-immunoperoxidase reaction using the Vectastain ABC kit (Vector Laboratories). Immunoreactivity was visualized by incubating the sections with 0.02% 3,3′-diaminobenzidine containing 0.01% hydrogen peroxide for 10 min. A purple-black reaction product was obtained by adding nickel chloride to the peroxidase reaction as previously described (Haxhiu, 1996). Stained sections were mounted on poly-L-lysine coated slides and immunoreactive cells were observed.

### Microscopy

The regions of the area postrema and the nucleus of the solitary tract were determined according to the brain map (Paxinos and Watson, 1998) as described previously (Horn, 2007). The caudal ventrolateral medullary region is caudal to the lateral reticular nucleus and regions enclosing the lateral reticular nucleus, and to the nucleus ambiguus (Wang et al., 2001; Stornetta et al., 2009). Images of brain areas with c-Fos immunoreactive cells were digitalized using a Canon EOS Kiss digital camera (Canon, Tokyo, Japan), with magnifications depending on the size and structure of each area. In addition, the number of c-Fos positive cells in the area postrema, the nucleus of the solitary tract, and the caudal ventrolateral medulla was counted using the NIH ImageJ software (National Institutes of Health, Bethesda, MD). Counting was performed by an observer who was blinded to the treatment groups.

### Data analysis

Statistical analysis of the data was performed using R software (R version 4.0.4; R Foundation for Statistical Computing, Vienna, Austria). After the Shapiro–Wilk test was used to evaluate normality, the values were expressed as the mean ± standard deviation. A paired *t*-test was used to compare changes in blood pressure before and after the stimulus, and Welch’s *t*-test was used for comparisons between groups. Tukey’s multiple comparison procedure was used to compare the total number of c-Fos immunoreactive cells in the area postrema, the nucleus of the solitary tract, and the caudal ventrolateral medulla. Differences were considered statistically significant at *p* < 0.05.

## Results

### Changes in blood pressure and heart rate with colorectal distension

**FIGURE 1:**
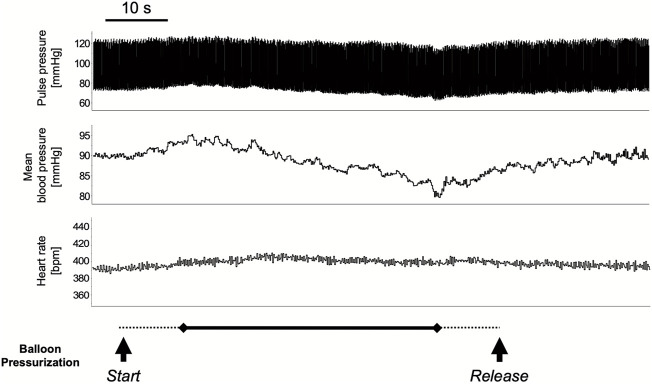
A typical response in circulatory dynamics with colorectal distension while balloon pressure is set at 80 mmHg. The distension procedure was performed by gradually increasing the target pressure for 10 s (dashed line), maintaining the pressure for 40 s (solid line), and then releasing it in 10 s (dashed line): for a total of 60 s. Mean arterial pressure falls immediately after the start of distension and gradually recovered after the release.

**FIGURE 2:**
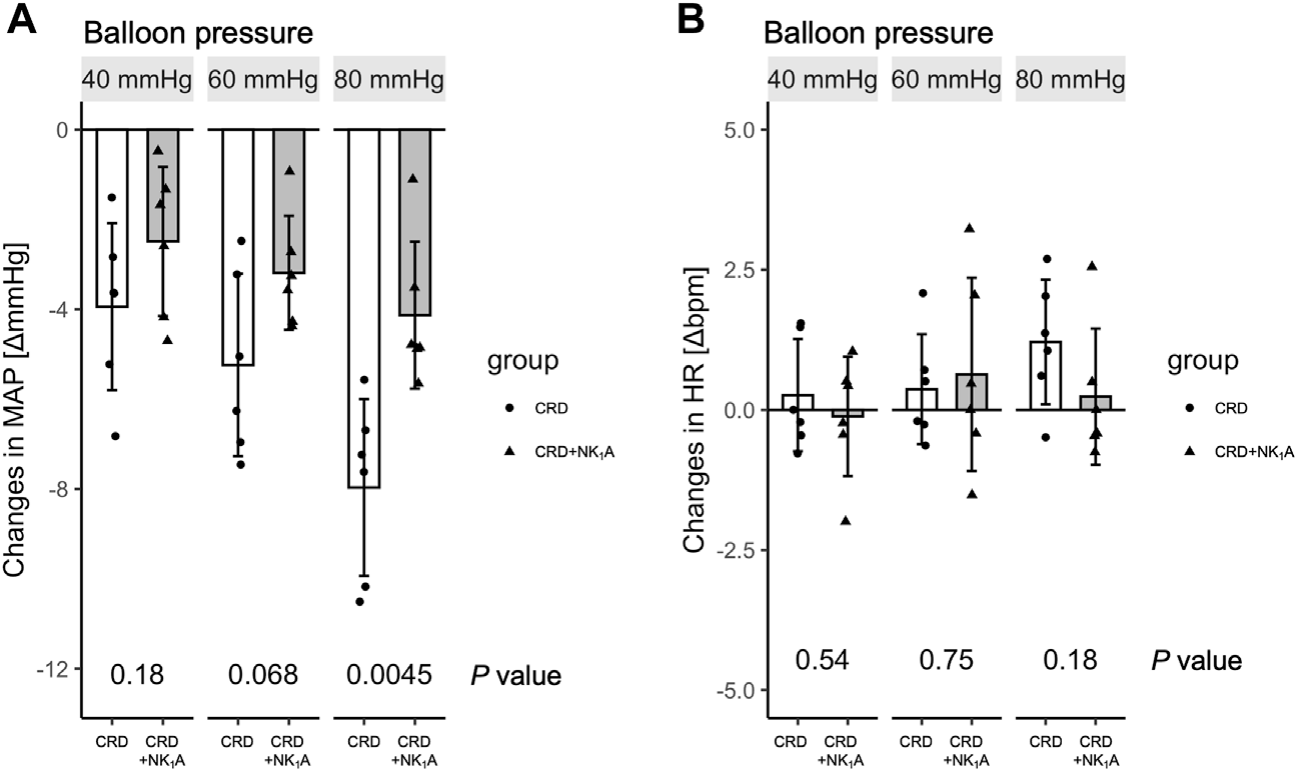
Changes in mean arterial pressure (MAP) and heart rate (HR) pre-and post-colorectal distension are shown in **(A)** and **(B)**, respectively. The distension procedure was performed at balloon pressures of 40, 60, and 80 mmHg. Group CRD received saline, and Group CRD + NK_1_A received fosaprepitant (30 mg/kg) intraperitoneally 40 min before the procedure. Data are expressed as mean ± standard deviation. *p* values: compared between groups using Welch’s *t*-test.

The colorectal distension procedure was performed at 40, 60, and 80 mmHg distension pressure. The distension procedure was performed by gradually increasing the target pressure for 10 s, maintaining the pressure for 40 s, and then releasing it in 10 s. The transient response in mean arterial pressure and heart rate is shown in Figure 1 with colorectal distension set at 80 mmHg: mean arterial pressure falls immediately after the start of distension and gradually recovers after the release of distension. Heart rate just before the start of the colorectal distension for Group CRD and CRD + NK_1_A was 431 ± 40 and 419 ± 30 bpm (*p* = 0.566), and mean arterial pressure was 89 ± 11 and 84 ± 11 mmHg (*p* = 0.466), respectively. In Group CRD, mean arterial pressure significantly decreased following stimulation in a pressure-dependent manner. In Group CRD + NK_1_A, the extent of the decrease in mean arterial pressure following stimulation with 80 mmHg was significantly less than that of Group CRD (Figure 2A). However, no difference in heart rate was observed between the groups (Figure 2B).

### Number of c-Fos immunoreactive cells in the area postrema, the nucleus of the solitary tract, and the caudal ventrolateral medulla

**FIGURE 3:**
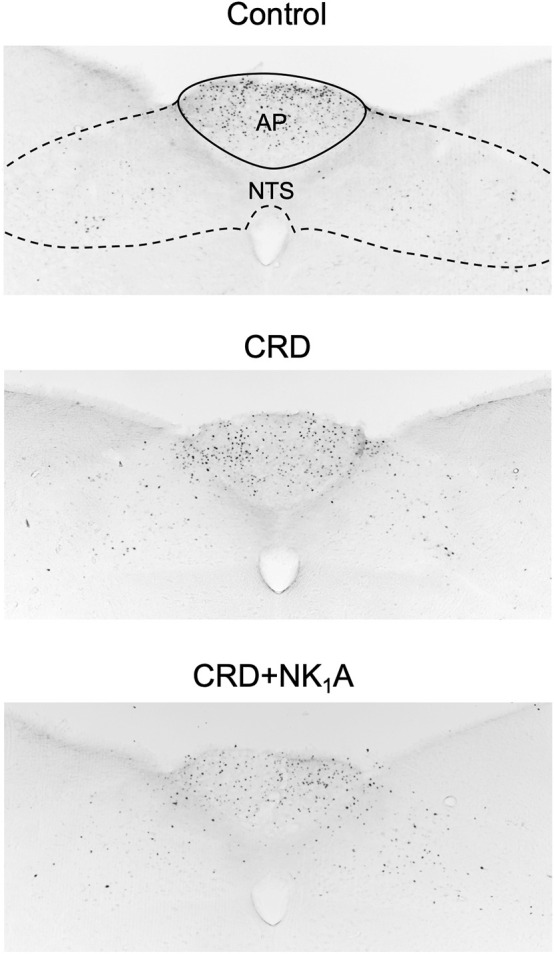
Representative vibratome sections through the area postrema (AP) and the nucleus of the solitary tract (NTS) from the groups stimulated by colorectal distension (Group CRD and CRD + NK_1_A) and non-stimulated (Control) rats. Group CRD received saline, and Group CRD + NK_1_A received fosaprepitant (30 mg/kg) intraperitoneally before the stimulation. The number of c-Fos immunoreactive cells in the NTS in the stimulated groups (CRD and CRD + NK_1_A) was higher than in the Control group.

**FIGURE 4:**
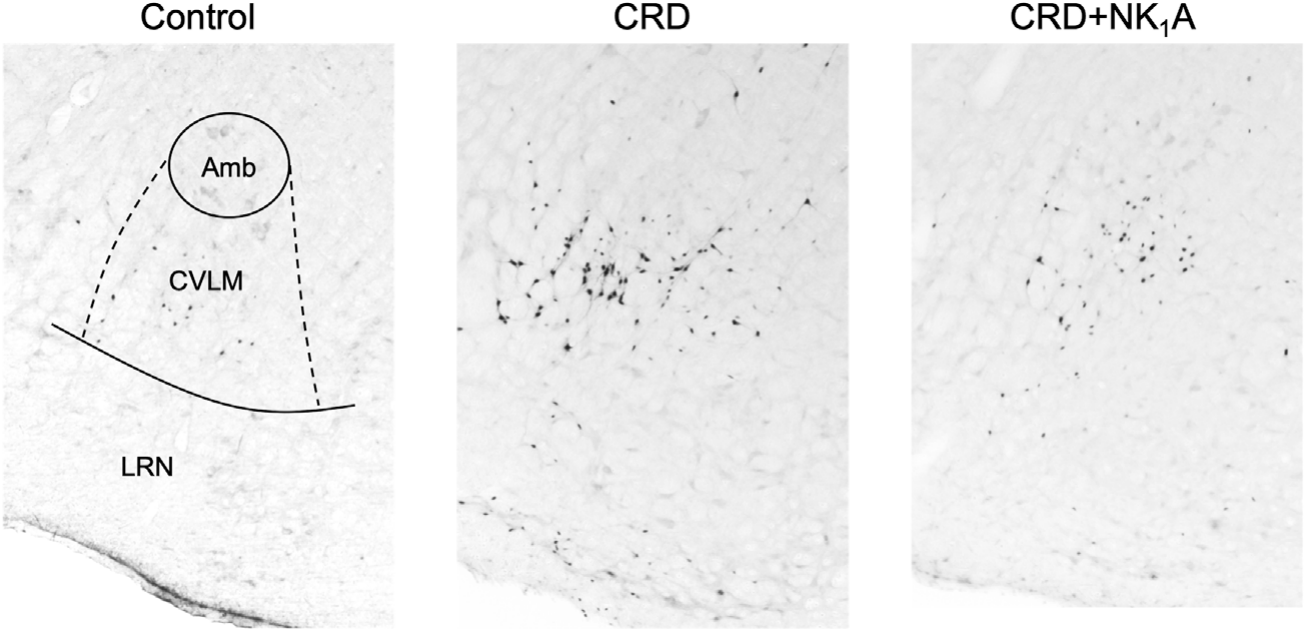
Representative vibratome sections through the caudal ventrolateral medulla (CVLM) from the groups stimulated by colorectal distension (Group CRD and CRD + NK_1_A) and non-stimulated (Control) rats. Group CRD received saline, and Group CRD + NK1A received fosaprepitant (30 mg/kg) intraperitoneally before the stimulation. The number of c-Fos immunoreactive cells in the CVLM in Group CRD was higher than in the Control group and Group CRD + NK_1_A. Amb: the nucleus ambiguus. LRN: the lateral reticular nucleus.

**FIGURE 5:**
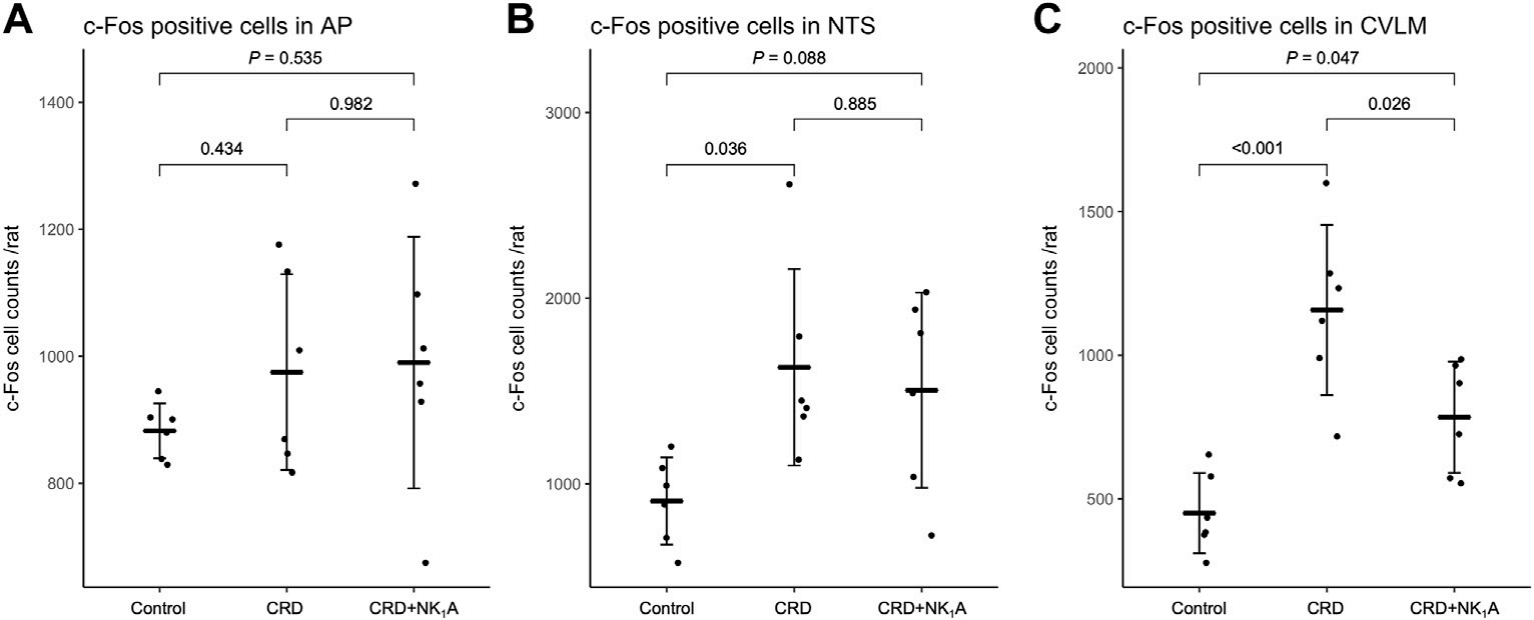
The number of c-Fos immunoreactive cells in AP **(A)**, NTS **(B)** and CVLM **(C)** from the groups stimulated by colorectal distension (Group CRD and CRD + NK_1_A) and non-stimulated (Control) rats. Group CRD received saline, and Group CRD + NK_1_A received fosaprepitant (30 mg/kg) intraperitoneally before the colorectal distension. Data are expressed as mean ± standard deviation. AP: the area postrema. NTS, the nucleus of the solitary tract; CVLM, the caudal ventrolateral medulla; *p* values: compared among groups using Tukey’s multiple comparison procedure.

The representative distributions of c-Fos immunoreactive cells in the area postrema and the nucleus of the solitary tract are shown in Figure 3, and the caudal ventrolateral medulla in Figure 4. In the area postrema, no difference was observed in the number of c-Fos positive cells among Group non-CRD, CRD, and CRD + NK_1_A (882 ± 43, 974 ± 154, and 990 ± 198 cells/rat, respectively: Figure 5A). In the nucleus of the solitary tract, the number of c-Fos positive cells in the non-CRD group was 907 ± 235 cells/rat, which was significantly increased in Group CRD (1,628 ± 530 cells/rat). There was no significant difference in the nucleus of the solitary tract between Group CRD and Group CRD + NK_1_A (1,628 ± 530 and 1,504 ± 526 cells/rat, respectively; Figure 5B). In the caudal ventrolateral medulla, the number of c-Fos positive cells in the control group was 450 ± 140 cells/rat and significantly increased after stimulation (Group CRD: 1,157 ± 296 cells/rat). Fosaprepitant administration markedly reduced the number of c-Fos positive cells in the caudal ventrolateral medulla (Group CRD + NK_1_A: 784 ± 194 cells/rat), and there was a significant difference between Group CRD and CRD + NK_1_A (Figure 5C).

## Discussion

The present study shows that colorectal distension induces a reduction in blood pressure as previously reported (Li et al., 2006), and that the administration of fosaprepitant at a dose of 30 mg/kg in rats could reduce the circulatory impact of the stimulation and lower neuroexcitation in the caudal ventrolateral medullary region in the vagal reflex pathway.

The proto-oncogene c-Fos can be used as a marker for neuronal activity (Greenberg et al., 1986). Therefore, immunohistochemical examination of c-Fos remains one of the most suitable methods for examining the active brain areas in experimental animals. For example, in the vagus stimulation rat model in a previous study, c-Fos protein accumulated in the regions associated with autonomic pathways but not in non-stimulated controls (Holstein et al., 2012).

The central pathways of the baroreceptor reflex have been outlined previously (Reis et al., 1984), and various neurotransmitters in the pre-sympathetic ganglion neurons of the ventrolateral medulla oblongata are considered candidates (Polson et al., 1992). Recently, the neurokinin receptor family has also been implicated in the central nervous system, which may play a role in cardiovascular regulatory mechanisms (Mistrova et al., 2016). Three peptides of this family, Substance-P, neurokinin-A, and neurokinin-B, have an established role as neurotransmitters in mammals (Maggi, 1995). Substance-P is the most abundant neuropeptide in the rat brain (Arai and Emson, 1986) and substantially contributes to central cardiovascular regulation and control of behavior (Itoi et al., 1992).

In a previous study, colorectal distension induced a reduction in blood pressure and renal sympathetic nerve activity in rats (Li et al., 2006). The present study showed an increased number of c-Fos immunoreactive cells due to colorectal distension in the nucleus of the solitary tract and caudal ventrolateral medulla; furthermore, these responses were suppressed by the neurokinin-1 receptor antagonist. This indicates that the neurokinin-1 receptors in the vagal reflex pathway may be involved in the response induced by colorectal distension. A previous study (Potts et al., 2007) reported that abrasion of the neurokinin-1 receptor-expressing nucleus of the solitary tract by Substance-P-conjugated toxin selectively abolished the depressive effect of somatosensory input on arterial baroreceptor-heart rate reflex function. This provides essential evidence for the action of neurokinin-1 receptors in the central nervous system. Another study (Culman et al., 1997) demonstrated that centrally administered neurokinin-1 receptor antagonists inhibit cardiovascular reactions in response to a noxious stimulus.

Several reports described the relationship between various visceral nerves and the central nervous system: such as the involvement of the colorectal vagus nerve with the area postrema (Zhao et al., 2022). On the contrary, the present study showed no increase in c-Fos positive cells in the area postrema due to colorectal distension. It is expected that the area postrema was not influenced as much by colorectal distension as other medullary regions. Furthermore, the administration of the neurokinin-1 receptor antagonists did not seem to affect c-Fos expression in the area postrema. This result might be related to the less distribution of the neurokinin-1 receptors in the area postrema compared with other sites in the medulla oblongata (Nakaya et al., 1994).

Our results in the stimulus groups showed no difference in the number of c-Fos positive cells in the area postrema and the nucleus of the solitary tract, with or without the neurokinin-1 receptor antagonist. In contrast, in the caudal ventrolateral medullary region, the administration of the neurokinin-1 receptor antagonist resulted in a decrease in the number of c-Fos positive cells. These phenomena indicate that the neurokinin-1 receptor antagonist inhibits the projection process from the nucleus of the solitary tract to the caudal ventrolateral medullary region. In other words, the present study showed that there were no differences in neural activity in the “input area” of the central nervous system, whereas there were differences in “inhibitory” neuronal activity, suggests that involvement of the reflex pathway at the neurokinin-1 receptors might take place in the medulla oblongata. It seems that various receptors and neuronal interrelationships might be involved in this phenomenon, and further studies are needed to clarify these details.

In conscious rats, colorectal distension stimulation induces a noxious visceral stimulus that produces cardiac responses such as tachycardia (Ness and Gebhart, 1988). However, no major changes in heart rate were detected in the present study. This might have occurred because anesthesia may have counteracted the visceromotor responses (Ness and Gebhart, 1988; Traub et al., 1995). Besides, mean blood pressure showed a slight increase in some rats at the beginning of stimulation, followed by a decrease. We believe this bi-phasic response was mediated by a sympathetic reflex *via* the spinal cord (Traub et al., 1992) and a subsequent parasympathetic reflex *via* the brain stem (Mönnikes et al., 2003). In addition, the cardiovascular response to colorectal distension depends on the type and depth of anesthesia, and the response is often variable (Ness and Gebhart, 1988). One might think, however, colorectal distension would induce a noxious visceral stimulus that causes in a decrease in blood pressure by inhibiting sympathetic output (Li et al., 2006) and the neurokinin-1 receptor antagonist, at least in part, influence the nociceptive reflex response. The involvement of such a neurokinin-1 receptor mechanism should be clarified in future studies.

Non-central nervous system actions of the neurokinin-1 receptor may also affect blood pressure; there are several reports in the periphery. Neurokinin-1 receptor antagonists may also have a neurokinin-A-blocking effect on peripheral sympathetic nerves, which may have locally suppressed the vascular endothelial response (Kaczynska et al., 2016). Another study in rats with colorectal distension showed that neurokinin-1 receptors mediate the rectocolonic inhibitory reflex, whereas neurokinin-A participates in visceral pain (Julia et al., 1994). In other words, neurokinin-1 receptors might be involved not only in vagal reflexes in the central nervous system but also in pain, enteroreceptors, and vascular smooth muscle.

In actual clinical practice, various phenomena that appear to be vagus nerve reflexes mediated by stimulant receptors of the gastrointestinal tract have been reported (Sadahiro et al., 1995; Neri et al., 2007; Bae et al., 2012; Kölükçü et al., 2019), but effective methods to control these reflexes have not been established. Suppression of such reflexes seems to be essential for safer clinical examination and treatment. The results of this study, which focused on the neurokinin-1 receptor, could provide new therapeutic possibilities for clinical medicine.

In conclusion, this study demonstrates that fosaprepitant suppresses the response induced by colorectal distension, and that it might be practical to treat the rectally stimulated reflex. Further studies are required to clarify the detailed distribution of receptors and to identify their relationships and effect.

## Data Availability

The original contributions presented in the study are included in the article, further inquiries can be directed to the corresponding author.

## Footnotes

1
https://blink.ucsd.edu/_files/sponsor-tab/iacuc/Urethane%20Guidelines%203-2016.pdf
